# Twice-Daily versus Once-Daily Pramipexole Extended Release Dosage Regimens in Parkinson's Disease

**DOI:** 10.1155/2017/8518929

**Published:** 2017-02-07

**Authors:** Ji Young Yun, Young Eun Kim, Hui-Jun Yang, Han-Joon Kim, Beomseok Jeon

**Affiliations:** ^1^Department of Neurology, Ewha Womans University Mokdong Hospital, Ewha Womans University College of Medicine, Seoul, Republic of Korea; ^2^Department of Neurology, Hallym University Sacred Heart Hospital, Hallym University College of Medicine, Anyang, Republic of Korea; ^3^Department of Neurology, Ulsan University Hospital, Ulsan, Republic of Korea; ^4^Department of Neurology and Movement Disorder Center, Seoul National University Hospital, Seoul National University College of Medicine, Seoul, Republic of Korea

## Abstract

This open-label study aimed to compare once-daily and twice-daily pramipexole extended release (PER) treatment in Parkinson's disease (PD). PD patients on dopamine agonist therapy, but with unsatisfactory control, were enrolled. Existing agonist doses were switched into equivalent PER doses. Subjects were consecutively enrolled into either once-daily-first or twice-daily-first groups and received the prescribed amount in one or two, respectively, daily doses for 8 weeks. For the second period, subjects switched regimens in a crossover manner. The forty-four patients completed a questionnaire requesting preference during their last visit. We measured the UPDRS-III, Hoehn and Yahr stages (H&Y) in medication-on state, Parkinson's disease sleep scale (PDSS), and Epworth Sleepiness Scale. Eighteen patients preferred a twice-daily regimen, 12 preferred a once-daily regimen, and 14 had no preference. After the trial, 14 subjects wanted to be on a once-daily regimen, 25 chose a twice-daily regimen, and 5 wanted to maintain the prestudy regimen. Main reasons for choosing the twice-daily regimen were decreased off-duration, more tolerable off-symptoms, and psychological stability. The mean UPDRS-III, H&Y, and PDSS were not different. Daytime sleepiness was significantly high in the once-daily regimen, whereas nocturnal hallucinations were more common in the twice-daily. Multiple dosing should be considered if once-daily dosing is unsatisfactory. This study is registered as NCT01515774 at ClinicalTrials.gov.

## 1. Introduction

The dopamine agonist pramipexole is an effective option for treatment of Parkinson's disease (PD) treatment [1, 2]. Pramipexole, when given in the immediate-release (IR) form, is taken orally three times a day. At a theoretical level, motor fluctuation risks can be reduced by continuous stimulation of the dopamine receptors [3, 4]. However, once-daily dosing has shown improved medication compliance [5] and less off-time [6]. To encourage stable dopaminergic delivery and compliance, an extended-release (ER) formulation of pramipexole was introduced.

The pramipexole extended-release (PER) formulation can be taken once daily and is reportedly not inferior to the IR formulation for efficacy, safety, and tolerability in early and advanced PD patients [7–9]. A once-daily PER dosage regimen permits smaller plasma concentration fluctuation and better convenience of use compared to those from a thrice-daily pramipexole IR (PIR) regimen [10].

However, we have met patients who express dissatisfaction with their motor fluctuations and adverse events (AEs) when taking once-daily PER, and some patients have asked for multiple daily dosing. In a previous study of a prolonged release form of ropinirole, we reported that multiple dosing might be a therapeutic option if once-daily dosing is unsatisfactory [11]. In this study, we evaluated preferences for dosing frequency in PER treatment and compared once-daily and twice-daily regimens by assessing a variety of measures of parkinsonism and sleepiness in patients with PD.

## 2. Methods

### 2.1. Patients

Inclusion criteria were patients with idiopathic PD, according to the United Kingdom Parkinson's Disease Society brain bank criteria [12], aged between 30 and 80 years old. All patients were on a dopamine agonist (PIR, ropinirole IR (RIR), or ropinirole ER (RER)) and were considering changing to PER due to suboptimal control with their current levodopa or dopamine agonist therapy. Reasons for changing to PER included subjective complaints of hypersomnolence, hallucination, motor fluctuation, drug-induced dyskinesia, and gastrointestinal discomfort. All patients were taking stable doses of antiparkinsonian medications including levodopa and dopamine agonist for at least 4 weeks prior to screening for inclusion in the study.

Patients with a history of significant or uncontrolled cognitive, neurologic, or other medical disorders; a history of impulsive compulsive disorder (ICD), depression, and apathy; a recent history or current evidence of drug abuse or alcoholism; severe dizziness or fainting due to orthostatic hypotension; a history of severe AEs related to dopaminergic agents; a history of allergic reaction to similar medications; or a history of heavy metal poisoning were eligible for enrollment. Patients were excluded if they were taking neuroleptics at the baseline visit. Patients were also excluded from the study if they had used PER or an investigational medication within the 4 weeks prior to study initiation. In addition, because a twice-daily dose could not be prescribed without splitting the tablet, patients who were on less than 0.7 mg of PIR or less than 3.0 mg of ropinirole were ineligible (Table 2).

### 2.2. Study Design

The investigation was designed as a 16-week, two-period, open-label crossover study to compare once-daily and twice-daily dosing of PER. The two 8-week crossover treatment periods were separated without a washout phase. The study protocol was approved by the Institutional Review Board of the Seoul National University Hospital (SNUH) and conformed to the principles of the Declaration of Helsinki. All patients signed an informed consent before participation in the study.

**Figure 1:**
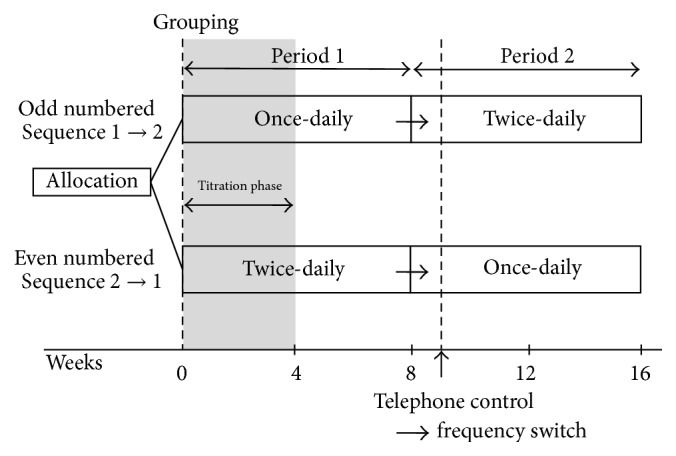
Study design.

Patients were arranged sequentially to once-daily dosing first or twice-daily dosing first groups in an open-label trial. Each group initially received 8 weeks of PER once daily or twice daily for 8 weeks and then switched to twice-daily or once-daily, respectively, for the final dosing schedule of 8 weeks. Crossover occurred without a washout period (Figure 1).

The conversion ratios between PIR to PER and RIR to PER were 1 : 1 and 5 : 1, respectively. Tablets of PER were available only in 0.375, 0.75, and 1.5 sizes; therefore, conversion from PIR or RIR to the PER dose was upwardly adjusted, if needed, to avoid breaking the PER tablets. For example, when PIR was being given at 1.1 mg/d, the PER dosage was 1.5 mg/d. In twice-daily dosing, we split the daily PER dose into two doses. If possible, the split doses were equal; however, unequal doses were used when necessary to avoid breaking PER (e.g., a 2.625 mg dose was converted to 1.5 mg and 1.125 mg doses, Table 2). First dosing of PER was given with the first dosing of the patient's other antiparkinsonian medications. The timing and dose of the second PER dosing was based on criteria they used to take dopaminergic agent prior to this study. Therefore, the second PER dosing occurred in the evening or the late afternoon and at an equal or lower dosage than the first PER dosing.

Titration of PER was undertaken only in the initial 4 weeks of the first period of the crossover sequence. The dose was titrated until an optimal therapeutic response was achieved or intolerable adverse effects disappeared. Dosing frequency was maintained during the titration phase. Once an optimal dose was achieved, the subject was maintained on that dose for the remainder of the study. Changes in dosage of other antiparkinsonian medications and sedatives were not allowed. In the second period of the crossover sequence, titration of PER was not allowed. However, if the subject complained of intolerable off-symptoms, dyskinesia, or adverse effects in the second period, early completion was accepted at a patient visit earlier than that at 16 weeks.

Patient visits to the clinic were scheduled to occur at baseline at weeks 1, 8, and 16. Telephone control was scheduled in week 9 (1 week after crossing over to second period) to check for AEs. Patients were allowed to make nonscheduled clinic visits when needed. At the baseline visit, all subjects were assessed by using the Unified Parkinson's Disease Rating Scale part 3 (mUPDRS) [13] and the Hoehn and Yahr stage (H&Y) [14] in the medication-on state, the Parkinson's Disease Sleep Questionnaire (PDSS) [15, 16], and the Epworth Sleepiness Scale (ESS) [17, 18].

At 8 and 16 weeks, or at last visit for early completion, all assessments were repeated and the Patient's Global Impressions (PGI) and compliance, including medication compliance, for each treatment period were determined. For each period, mean compliance rates were calculated based on the total prescribed doses and total actually taken doses for all subjects. In addition, AEs and changes in wearing-off and dyskinesia were recorded throughout the study.

At the completion of the second period, patients' dosage regimen preference was assessed by asking the following question: “Which regimen do you prefer?” The possible answers were “I prefer the once-daily dosing,” “I prefer the twice-daily dosing,” and “I do not prefer one regimen over the other.” If subjects completed the study earlier than the end of the second treatment period, they were still asked for their preferences. Patients who did not complete the first treatment period or did not complete the preference questionnaire were excluded from the analysis.

In addition, patients' medication choices after completing this trial were also determined by asking the question: “Which regimen will you choose after this trial?” Possible choices were “PER” and “other dopamine agonists.” If they chose “PER,” we asked them “which PER regimen will you choose between once-daily and twice-daily dosing?” In addition, we asked for their reasons for their choices.

### 2.3. Outcome Measures

The primary outcome measure was the preference of the subjects for once-daily or twice-daily PER treatment as reported at study completion or at early completion after crossover. Secondary outcome measures included the mUPDRS [13], H&Y [14] in the medication-on state, PDSS [15, 16], ESS [17, 18], compliance, and the proportion of PGI of improvement (PGI-I). In addition, the duration and severity of motor complication were evaluated by using visual rating scales for wearing-off and dyskinesia, respectively.

To monitor patient safety, AEs were recorded throughout the study. We assessed the occurrence, type, and intensity of AEs. The intensity of AEs was categorized by severity as mild (causing minimal discomfort and not interfering with everyday activities), moderate (sufficiently discomforting to interfere with normal daily activities), or severe (incapacitating or causing inability to undertake usual activities). A serious AE was defined as fatal, life-threatening, required or prolonging hospitalization, or leading to significant disability.

### 2.4. Statistical Analyses

Comparisons of the once-daily to twice-daily (1 → 2) and twice-daily to once-daily (2 → 1) crossover groups at baseline were performed by using Mann-Whitney tests. The primary analysis was based on descriptive statistics to determine subject preferences. Comparisons of the groups that preferred once-daily or twice-daily were analyzed by applying Mann-Whitney tests. Comparisons of mUPDRS, H&Y, PGI-I, PDSS, ESS, compliance, and visual analogue scale (VAS) for wearing-off and dyskinesia values for the once-daily and twice-daily periods were analyzed by performing Wilcoxon signed rank tests. McNemar tests were used to evaluate differences in AE occurrence in the once-daily and twice-daily periods. Statistical analyses were done by using SPSS statistical package version 21 (SPSS Inc., Chicago, IL, USA).

## 3. Results

### 3.1. Subjects and Subject Discontinuations

**Figure 2:**
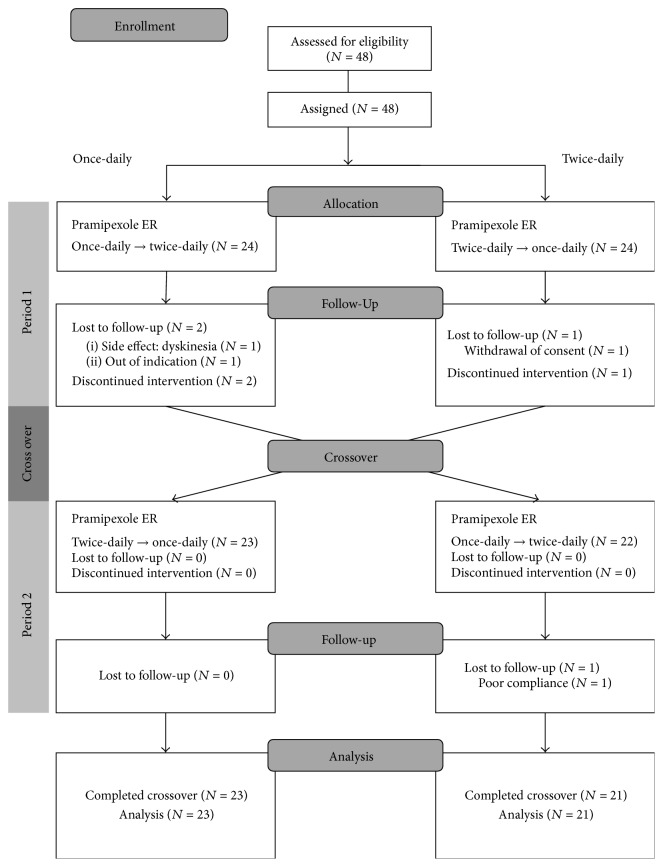
Subject flow chart.

Forty-eight patients with PD were enrolled in this study. Of those, 24 patients started the once-daily regimen and another 24 patients the twice-a-day regimen. Four subjects (8.3%) did not complete the study (Figure 2). Three patients discontinued the study during sequence 1 → 2 and one patient discontinued during sequence 2 → 1. One patient was excluded after self-titrating her levodopa dose without noticing in the once-daily regimen. According to dosing frequency, two subjects discontinued in each dosing regimen. According to treatment period, three patients dropped out during the first period and one patient discontinued during the second period. As a result, 44 subjects (91.7% of those enrolled) were included in the final analysis (Figure 2). Baseline demographics characteristics were not significantly different between those two treatment groups (Table 1).

At the baseline visit, one patient was on RIR (6 mg/day), 20 patients on RER (10.6 ± 6.4 mg/day), and 27 on PIR (2.2 ± 1.1 mg/day). Based on the study's conversion ratios, the dose levels of RIR, RER, and PIR averaged 2.2 ± 1.2 mg/day of PER. To avoid breaking the PER tablets, the average actual converted dose of PER was 2.4 ± 1.2 mg/day before titration (Table 2). After the titration, the average dose of PER was 2.5 ± 1.2 mg/day.

### 3.2. Primary and Secondary Outcomes

**Figure 3:**
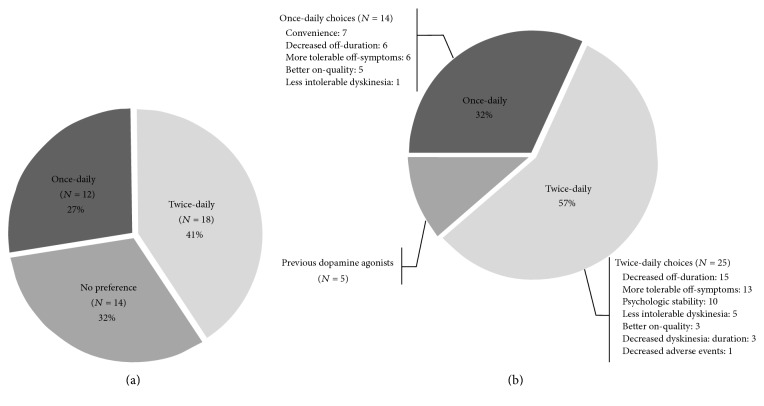
Patient's preferences and choices in the end of the trial. (a) Preferences: in response to the final preference questionnaire, 27% (*n* = 12) of the patients preferred the once-daily regimen, 41% (*n* = 18) preferred the twice-daily regimen, and 32% (*n* = 14) did not have a preference. (b) Patient's choices in the end of the trial and reasons. In response to the final choices questionnaire, 32% (*n* = 14) of the patients wanted to take the once-daily regimen, 57% (*n* = 25) chose the twice-daily regimen, and 11% (*n* = 5) wanted to revert to previous dopamine agonists.

Analysis of responses to the preference questionnaire showed that 27% (*n* = 12) of the patients preferred the once-daily regimens, 41% (*n* = 18) preferred the twice-daily regimens, and 32% (*n* = 14) did not express a preference for either regimen (Figure 3(a)).

Their mean ESS was higher in once-daily group than in the twice-daily group (4.8 ± 2.9 versus 4.3 ± 2.8,* P* = 0.040). However, the mean mUPDRS, H&Y, PGI-I, compliance, and AE values were not significantly different between the two regimens (Table 3). Total PDSS tended to be higher in twice-daily group; however the difference was not significant (*P* = 0.082). With regard to PDSS subscores, the night hallucinations were more common in the twice-daily group (*P* = 0.008). At the end of each period, the proportions of PGI-I were not significantly different between the two regimens (*P* = 0.279, Table 3).

With regard to wearing-off and dyskinesia, the VAS scores for duration and severity were not significantly different between the once-daily and twice-daily regimens (*P* = 0.872 and* P* = 0.284, resp., for wearing-off;* P* = 0.690 and* P* = 0.472, resp., for dyskinesia). Although the PGI-I tended to be higher in twice-daily group than the once-daily group, there was no significant difference between PGI-I values (*P* = 0.109).

### 3.3. Patients' Choices at Completion

Analysis of the responses to the questionnaire about patients' choices at completion of the clinical trial showed that 39 patients chose to remain on PER; five patients chose to revert to the other dopamine agonists that they had taken previous to the trial. Among patients whose choice was to continue with PER, 32% (*n* = 14) wanted to follow a once-daily regimen, whereas 57% (*n* = 25) chose the twice-daily regimens (Figure 3(b)).

Among the patients that chose the once-daily regimen, the reasons provided were convenience (*n* = 7), decreased off-duration (*n* = 6), more tolerable off-symptoms (*n* = 6), better on-quality (*n* = 5), and less intolerable dyskinesia (*n* = 1). The reasons for choosing the twice-daily regimen were decreased off-duration (*n* = 15), more tolerable off-symptoms (*n* = 13), psychological stability (*n* = 10), less intolerable dyskinesia (*n* = 5), better on-quality (*n* = 3), decreased dyskinesia duration (*n* = 3), and decreased AEs (*n* = 1).

Among the 5 patients who choose to revert to the previous dopamine agonist treatment, 3 patients had taken PIR thrice-daily and 2 patients took RER twice-daily. The reasons for their choices were more tolerable off-symptoms (*n* = 3, two of RER and one PIR patients), more tolerable dyskinesia (1 PIR patient), and psychological stability due to thrice-daily dosing (1 PIR patient).

Additionally, we compared baseline characteristics between the groups who wanted to be on the once-daily regimen and those who wanted to follow the twice-daily regimen after completing the trial (Table e-1 in Supplementary Material available online at https://doi.org/10.1155/2017/8518929). The patients who chose the twice-daily regimen had longer disease duration (*P* = 0.005) and a longer wearing-off duration (*P* = 0.047). In addition, they had more severe difficulty in staying asleep, more frequent getting-up related to voiding, and more severe daytime sleepiness (*P* = 0.047,* P* = 0.035, and* P* = 0.039, resp.). There was no statistically significant difference between the baseline nocturnal hallucination values between those who chose once-daily and twice-daily regimens.

### 3.4. Adverse Events

The incidence of drug-related AEs did not differ significantly between the once-daily (81.8%, 36/44) and twice-daily (84.1%, 37/44) regimens (*P* = 1.000, Table 4). The most common drug-related AE in both regimens was constipation. Impulsive compulsive behavior, depression, and apathy related to dopamine agonist were not reported in our study.

In the twice-daily regimen, five patients complained the nocturnal visual hallucinations as AEs. Two patients had the visual hallucinations with both regimens. Their intensity of hallucination was mild; however, they complained the hallucinations were more vivid with the twice-daily regimen.

## 4. Discussion

Generally, PER is prescribed for use in a once-daily regimen. In our assessment of PER in once-daily and twice-daily regimens, when patients switched from once-daily to twice-daily or twice-daily to once-daily regimens their mean mUPDRS, H&Y, ESS, and PDSS and AEs values did not change significantly. Despite the different dosing frequency, the results indicate that whether the daily dosage of PER is provided as one dose or split into two doses, the two treatment regimens have similar efficacy and no difference in the incidences of AEs.

In a previous study, patients had a preference for once-daily PER rather than thrice-daily PIR [19]. In our study, 27% (*n* = 12) of the patients preferred the once-daily regimens, and 32% (*n* = 14) of the patients preferred a once-daily regimen. Among the patients that chose to follow the once-daily regimen, the commonest reasons for that choice were convenience, decreased off-duration, and more tolerable off-symptoms in our study.

In this study, a greater percentage of patients chose to adopt a twice-daily regimen rather than a once-daily regimen after the trial (57 % and 32%, resp.). Among patients who chose the twice-daily regimen, the commonest reasons were decreased off-duration, more tolerable off-symptoms, and psychological stability.

Among the 5 patients that chose to revert to their previous dopaminergic therapy with multiple dosing, the commonest reason for that choice was also more tolerable off-symptoms (*n* = 3). In addition, one patient chose thrice-daily dosing to achieve psychological comfort. The results indicate that anxiety about off-symptoms may contribute to a patient's dosage regiment reference.

Sleepiness and hallucination are recognized adverse effects of dopamine agonists, including PER [20–22]. In our study, daytime sleepiness was significantly severe in the once-daily group compared to that in the twice-daily group, whereas nocturnal hallucinations were more common in the twice-daily group than in the once-daily group. Thus, clinicians should consider a twice-daily regimen for patients who complain of daytime sleepiness and a once-daily regimen for patients with nocturnal hallucinations.

Among patients taking antiparkinsonian medication, once-daily dosing may improve compliance [23]. In our study, there was no significant difference in compliance levels between once-daily and twice-daily regimens. Because many PD patients are taking multiple antiparkinsonian medications many times a day already, compliance may not be a critical issue, especially for patients with advanced PD.

This study was an open-label study, and there were few statistically significant differences between the once-daily and twice-daily dosage regimen group. Therefore, if patients currently on once-daily PER are not satisfied with that regimen, due to off-symptoms, AEs, sleep-related symptoms, or anxiety related to off-symptoms, clinicians should consider trying a twice-daily dosage regimen.

## Supplementary Material

Supplementary table e-1. Comparisons of baseline characteristics between the patients who chose once-daily regimens and twice- daily after the trial.

## Figures and Tables

**Table 1:** Baseline characteristics in the per-protocol population.

	QD → BID (*N* = 21)	BID → QD (*N* = 23)	*P* Value	Overall (*N* = 44)
Age (years)	62.3 ± 8.7	58.4 ± 8.8	0.095	60.3 ± 8.9
Disease duration	8.9 ± 5.0	10.7 ± 5.4	0.187	9.8 ± 5.3
Sex (M : F)	10 : 11	9 : 14	0.570	19 : 25
mUPDRS	19.7 ± 7.6	17.3 ± 7.9	0.365	18.5 ± 7.7
Hoehn and Yahr stage	2.1 ± 0.4	2.0 ± 0.5	0.728	2.0 ± 0.4
Pramipexole ER dose before titration	2.4 ± 1.2	2.3 ± 1.2	0.943	2.4 ± 1.2
Pramipexole ER dose after titration	2.5 ± 1.3	2.4 ± 1.2	0.925	2.5 ± 1.2
LEDD	937.5 ± 323.8	1036.1 ± 252.3	0.455	989.0 ± 289.5
Epworth Sleep Scale	4.5 ± 3.3	5.0 ± 2.7	0.463	4.8 ± 3.0
PDSS				
Overall sleep quality	7.7 ± 2.3	7.2 ± 2.0	0.331	7.5 ± 2.2
Falling in sleep	8.5 ± 2.9	8.2 ± 2.3	0.348	8.3 ± 2.6
Staying asleep	5.5 ± 4.6	4.7 ± 4.1	0.457	5.1 ± 4.3
Sleep disruption due to restlessness of limbs at night or evening	8.8 ± 2.2	8.3 ± 3.2	0.749	8.5 ± 2.7
Fidget in bed	7.9 ± 3.5	8.7 ± 2.0	0.728	8.3 ± 2.8
Distressing dreams at night	8.1 ± 3.6	8.2 ± 2.9	0.668	8.2 ± 3.2
Distressing hallucination at night	9.0 ± 2.4	9.1 ± 2.5	0.641	9.0 ± 2.4
Getting up at night to pass urine	4.1 ± 4.7	4.2 ± 4.3	0.930	4.2 ± 4.5
Incontinence due to off-symptoms	10.0 ± 0.0	10.0 ± 0.0	1.000	10.0 ± 0.0
Numbness or tingling of limbs	8.3 ± 3.1	8.8 ± 2.3	0.945	8.6 ± 2.7
Painful muscle cramps	8.3 ± 2.8	9.0 ± 2.2	0.680	8.7 ± 2.5
Wake early in the morning with painful posturing of limbs	9.6 ± 1.7	9.6 ± 0.9	0.224	9.6 ± 1.4
On waking tremor	8.9 ± 2.8	8.8 ± 3.1	0.958	8.8 ± 2.9
Morning tiredness or sleepiness	7.9 ± 3.8	8.4 ± 3.0	0.932	8.2 ± 3.4
Unexpected falling asleep in the day	8.4 ± 2.6	8.6 ± 2.7	0.785	8.5 ± 2.6
Total PDSS	121.0 ± 24.4	121.9 ± 16.1	0.589	121.5 ± 20.2
VAS for wearing off-duration	7.8 ± 1.5	7.6 ± 1.3	0.524	7.7 ± 1.4
VAS for wearing off-severity	6.2 ± 2.6	6.0 ± 1.5	0.569	6.1 ± 2.1
VAS for dyskinesia-duration	9.1 ± 1.1	8.5 ± 2.2	0.500	8.8 ± 1.8
VAS for dyskinesia-severity	8.3 ± 2.2	8.6 ± 1.7	0.941	8.4 ± 2.0

QD, once-daily; BID, twice-daily; mUPDRS, Unified Parkinson's Disease Rating Scale part 3; ER, extended-release; LEDD, levodopa equivalent dose; VAS, visual analogue scale; PDSS, Parkinson's disease sleep scale.

**Table 2:** Dose switches from conventional dopaminergic agonist to pramipexole ER.

Pramipexole IR → pramipexole ER			Ropinirole → pramipexole ER		
Pramipexole IR daily dose, mg	Pramipexole ER, mg		Ropinirole daily dose, mg	Pramipexole ER, mg	
	Once-daily	Twice-daily		Once-daily	Twice-daily
0.7 ≤ pramipexole < 0.8	0.75	0.375-0.375	3.0 ≤ ropinirole < 4.0	0.75	0.375-0.375
0.8 ≤ pramipexole < 1.1	1.125	0.75-0.375	4.0 ≤ ropinirole < 7.0	1.125	0.75-0.375
1.1 ≤ pramipexole < 1.6	1.5	0.75-0.75	7.0 ≤ ropinirole < 8.0	1.5	0.75-0.75
1.6 ≤ pramipexole < 1.9	1.875	1.125-0.75	8.0 ≤ ropinirole < 10.0	1.875	1.125-0.75
1.9 ≤ pramipexole < 2.3	2.25	1.125-1.125	10.0 ≤ ropinirole < 12.0	2.25	1.125-1.125
2.3 ≤ pramipexole < 2.7	2.625	1.5-1.125	12.0 ≤ ropinirole < 14.0	2.625	1.5-1.125
2.7 ≤ pramipexole < 3.1	3.0	1.5-1.5	14.0 ≤ ropinirole < 16.0	3.0	1.5-1.5
3.1 ≤ pramipexole < 3.4	3.375	1.875-1.5	16.0 ≤ ropinirole < 17.0	3.375	1.875-1.5
3.4 ≤ pramipexole < 3.8	3.75	1.875-1.875	17.0 ≤ ropinirole < 19.0	3.75	1.875-1.875
3.8 ≤ pramipexole < 4.2	4.125	2.25-1.875	19.0 ≤ ropinirole < 21.0	4.125	2.25-1.875
4.2 ≤ pramipexole < 4.6	4.5	2.25-2.25	21.0 ≤ ropinirole < 23.0	4.5	2.25-2.25
4.6 ≤ pramipexole < 4.9	4.875	2.625-2.25	23.0 ≤ ropinirole < 25.0	4.875	2.625-2.25

IR, immediate-release; ER, extended-release.

**Table 3:** Secondary outcomes in the per-protocol population.

	Once-daily	Twice-daily	*P* value
mUPDRS	18.1 ± 8.2	17.8 ± 7.8	0.830
Hoehn and Yahr stage	2.0 ± 0.5	2.0 ± 0.4	0.655
Compliance (%)	99.4 ± 1.6	99.7 ± 0.9	0.182
Total sleep time (hours)	5.5 ± 1.3	5.6 ± 1.4	0.134
Epworth Sleep Scale	4.8 ± 2.9	4.3 ± 2.8	0.040^a^
PDSS			
Overall sleep quality	7.6 ± 2.1	8.2 ± 1.6	0.061
Falling in sleep	8.4 ± 2.5	8.8 ± 2.1	0.234
Staying asleep	5.4 ± 4.3	5.9 ± 4.0	0.347
Sleep disruption due to restlessness of limbs at night or evening	8.4 ± 2.9	8.7 ± 2.9	0.100
Fidget in bed	8.6 ± 2.9	9.0 ± 2.5	0.504
Distressing dreams at night	8.6 ± 2.9	8.7 ± 2.6	0.550
Distressing hallucination at night	9.6 ± 1.4	8.6 ± 3.0	0.008^a^
Getting up at night to pass urine	3.4 ± 4.5	3.9 ± 4.3	0.314
Incontinence due to off-symptoms	9.8 ± 1.5	10.0 ± 0.0	0.317
Numbness or tingling of limbs	8.8 ± 2.2	8.7 ± 2.7	0.555
Painful muscle cramps	8.7 ± 2.3	8.8 ± 2.2	0.825
Wake early in the morning with painful posturing of limbs	9.2 ± 2.2	9.3 ± 2.2	0.859
On waking tremor	9.4 ± 2.1	9.2 ± 2.3	0.285
Morning tiredness or sleepiness	7.8 ± 3.6	8.0 ± 3.1	0.875
Unexpected falling asleep in the day	8.5 ± 2.3	8.7 ± 2.2	0.281
Total PDSS	122.3 ± 18.1	124.4 ± 19.1	0.082
VAS for wearing off-duration	8.1 ± 1.3	8.1 ± 1.3	0.872
VAS for wearing off-severity	6.2 ± 2.2	6.3 ± 2.2	0.284
VAS for dyskinesia-duration	9.1 ± 1.7	9.1 ± 1.6	0.690
VAS for dyskinesia-severity	8.6 ± 2.0	8.6 ± 1.9	0.472
PGI-I, no (%)	17 (38.6)	23 (52.3)	0.180

^a^ *P* < 0.05.

mUPDRS, Unified Parkinson's Disease Rating Scale part 3; VAS, visual analogue scale; PDSS, Parkinson's disease sleep scale; PGI-I, patient global impressions of improvement.

**Table 4:** Adverse events.

	Baseline	Once-daily	Twice-daily
Adverse events (%)	36 (81.8)	36 (81.8)	37 (84.1)
Constipation	23 (52.3)	24 (54.5)	20 (45.5)
Dry mouth	19 (43.2)	20 (45.5)	22 (50.0)
Somnolence	11 (25.0)	12 (27.3)	11 (25.0)
Dizziness	9 (20.5)	8 (18.2)	8 (18.2)
Fatigue	8 (18.2)	8 (18.2)	9 (20.5)
Dyspepsia	7 (15.9)	8 (18.2)	10 (22.7)
Nausea	7 (15.9)	6 (13.6)	7 (15.9)
Edema	5 (11.4)	7 (15.9)	9 (20.5)
Hallucination	3 (6.8)	2 (4.5)	5 (11.4)
Headache	2 (4.5)	2 (4.5)	1 (2.3)
Others	2 (4.5)	0 (0.0)	0 (0.0)
