# Supplementary material for: Twice-Daily versus Once-Daily Pramipexole Extended Release Dosage Regimens in Parkinson's Disease
Source: Parkinsons Dis. 2017 Feb 7;2017:8518929. doi: 10.1155/2017/8518929 (PMC5318624; doi:10.1155/2017/8518929)
Supplement: Supplementary file 1 — Supplementary table e-1. Comparisons of baseline characteristics between the patients who chose once-daily regimens and twice- daily after the trial. [file 8518929.f1.pdf]

Supplementary table e-1. Comparisons of baseline characteristics between the patients who chose once-daily regimens and twice- daily after the trial.

N = 39	QD (N = 14)	BID (N = 25)	*P* Value
Age (years)	58.0 ± 11.6	61.1 ± 7.1	0.792
Disease duration	7.7 ± 5.9	11.2 ± 4.9	0.005[a]
Sex (M:F)	5:9	11:14	0.637
mUPDRS	18.8 ± 10.1	19.0 ± 6.5	0.792
Hoehn and Yahr stage	1.9 ± 0.6	2.1 ± 0.4	0.157
Pramipexole ER dose after titration	2.0 ± 1.0	2.7 ± 1.3	0.137
LEDD	966.3 ± 330.8	970.1 ± 256.3	0.638
Epworth Sleep Scale	3.2 ± 2.0	5.4 ± 3.3	0.039[a]
PDSS			
Overall sleep quality	7.8 ± 2.2	7.1 ± 2.2	0.332
Falling in sleep	8.4 ± 3.5	8.4 ± 2.2	0.285
Staying asleep	7.1 ± 4.1	4.5 ± 4.1	0.042[a]
Sleep disruption due to restlessness of limbs at night or evening	8.3 ± 3.2	8.3 ± 2.7	0.767
Fidget in bed	8.0 ± 3.1	8.4 ± 2.9	0.474
Distressing dreams at night	8.3 ± 3.6	7.9 ± 3.3	0.599
Distressing hallucination at night	9.3 ± 2.0	9.1 ± 2.3	0.481
Getting up at night to pass urine	6.3 ± 3.8	3.8 ± 4.1	0.035
Incontinence due to off symptoms	10.0 ± 0.0	10.0 ± 0.0	1.000
Numbness or tingling of limbs	7.5 ± 3.5	8.9 ± 2.3	0.133
Painful muscle cramps	9.4 ± 1.2	8.4 ± 2.9	0.591
Wake early in the morning with painful posturing of limbs	9.8 ± 0.7	9.5 ± 1.6	0.656
On waking tremor	7.6 ± 4.0	9.3 ± 2.2	0.068
Morning tireness or sleepiness	9.1 ± 2.7	7.5 ± 3.8	0.119
Unexpected falling asleep in the day	9.7 ± 0.7	8.1 ± 3.0	0.073
Total PDSS	126.5 ± 23.0	119.3 ± 20.0	0.208
VAS for wearing off-duration	2.0 ± 1.1	2.7 ± 1.3	0.115
VAS for wearing off-severity	6.6 ± 2.1	5.8 ± 2.2	0.288

VAS for dyskinesia-duration	9.4 ± 0.7	8.6 ± 1.8	0.291
VAS for dyskinesia-severity	8.5 ± 1.8	8.3 ± 2.2	0.975

[a]P <0.05

QD, Once-daily; BID, Twice-daily; mUPDRS, Unified Parkinson's Disease Rating Scale part 3; ER, extended-release; LEDD, levodopa equivalent dose; VAS, visual analogue scale; PDSS, Parkinson's disease sleep scale.
